# Impact of COVID-19 effective reproductive rate on cryptocurrency

**DOI:** 10.1186/s40854-022-00354-5

**Published:** 2022-05-16

**Authors:** Marcel C. Minutolo, Werner Kristjanpoller, Prakash Dheeriya

**Affiliations:** 1 Robert Morris University, 6001 University Blvd, Moon Township, PA 15108 USA; 2 Departamento de Industrias, Universidad Técnica Federico Santa María, Av. España 1680, Valparaíso, Chile; 3 California State University Dominguez Hills, 1000 E Victoria St, Carson, CA 90747 USA

**Keywords:** COVID-19, Cryptocurrency, Bitcoin, GARCH, Digital currency, Fintech

## Abstract

The importance of cryptocurrency to the global economy is increasing steadily, which is evidenced by a total market capitalization of over $2.18T as of December 17, 2021, according to coinmarketcap.com (Coin, 2021). Cryptocurrencies are too confusing for laymen and require more investigation. In this study, we analyze the impact that the effective reproductive rate, an epidemiological indicator of the spread of COVID-19, has on both the price and trading volume of eight of the largest digital currencies—Bitcoin, Ethereum, Tether, Ripple, Litecoin, Bitcoin Cash, Cardano, and Binance. We hypothesize that as the rate of spread decreases, the trading price of the digital currency increases. Using Generalized Autoregressive Conditional Heteroskedasticity models, we find that the impact of the spread of COVID-19 on the price and trading volume of cryptocurrencies varies by currency and region. These findings offer novel insight into the cryptocurrency market and the impact that the viral spread of COVID-19 has on the value of the major cryptocurrencies.

## Introduction

The “COVID” year (2020) has seen a great deal of turbulence worldwide. Economies across the world have been negatively impacted—unemployment soared, work was done remotely, quarantine protocols kept people from going out, and supply chains were disrupted. The turbulence exposed firms to greater financial risk. Kou et al. (2014) stated that financial risk uncertainties affect all forms of financing. Amidst the pandemic, cryptocurrencies (hereafter CCs) also experienced a great deal of volatility. For instance, on March 1, 2020, Bitcoin was trading at a mere $8,562 per coin, and within a year, on March 7, 2021, it climbed to $51,207.

In a recent special issue on the impact of COVID-19 on CCs, Xiao et al. (2021) commented on the sharp rise of Bitcoin, especially during the COVID-19 pandemic. In that same special issue, Sebastião and Godinho (2021) applied machine learning to predict three of the major CCs but opened with the question “What is Bitcoin?” Cohen (2017) argued that CCs are a form of “weak currency,” which is characterized by a lack of investor incentives to accumulate the currency. However, Gajardo et al. (2018) noted that CCs are rapidly entering the global financial market and gaining importance. Based on the growth of the value of CCs within a year, one might argue that the rise in its importance in global financial markets could be due to the COVID-19 pandemic. We theorize that the rise in the price of CC is because of the ease with which CC can be moved around the world, as peoples’ movements were restricted during this period. The rise in the value of Bitcoin, the most popular CC, may be accounted for by the fact that it is near frictionless to transfer from one location in the world to another. This raises the following question: “Has CC become what Cohen (2017) referred to as a ‘strong currency’,” one that incentivizes people to leverage it for financial gain and a hedge against economic risk?

Lim et al. (2016) reported on two rulings by the Commodity Futures Trading Commission that found that virtual currencies are commodities subject to the Commodity Exchange Act. If it has become a “strong currency,” then what are its managerial implications? Furthermore, in general, if CCs demonstrate patterns of club convergence, as found with Bitcoin (Sahoo 2020), and are “near stock” (Sahoo et al. 2019), we expect them to perform similarly to each other (club dynamics) and the market (near stock). Because some have suggested that CCs may be used as a hedge investment (Dyhrberg 2016b), it is important to understand how they react during crises. Thus, we are motivated to measure the impact that the spread of the COVID-19 pandemic has on the price of major CCs and the subsequent investment strategies thereof.

To put the CC market in context, the total market capitalization of all CCs was over $ 2.18 trillion around the middle of December 2021, and there are over 16,600 CCs. Out of the total market for CC, Bitcoin accounts for approximately 40.9% ($781.4 billion at the time of writing this paper) and Ethereum (ETH), the second largest, 18.9% ($360.5 billion). The drop in the price of Bitcoin alone from $51,207 on March 7, 2021 to $29,807 on July 19, 2021 (the one-year low) amounted to a drop of over $500 billion in its total market valuation. In this study, we have adopted the convention in the computer science literature to use “Bitcoin” (capital letter B) to refer to the system and bitcoin (small letter b) to refer to the unit of account (Böhme et al. 2015).

The remainder of this paper is structured as follows. In the next section, we provide a brief literature review of CC. We then present our methods, which include the data used, where they were sourced from, and the approach used to analyze them. Then, we present the results of the models. Finally, we discuss the research and managerial implications of the study.

### Literature review

Kou et al. (2021) stated that financial technology has the potential to improve banking by reducing costs and improving customer satisfaction. Blockchain and CC are financial technologies to help in this regard. Since the introduction of blockchain and CC, much research has been conducted to understand their nature. We refer to the paper of Narayanan et al. (2016) for a good review of Bitcoin, blockchain technology, and the CC environment. Noga (2017) presented a good discussion of the role of money and alternative currencies. Finally, Conti et al. (2018) and Tschorsch and Scheuermann (2016) provided a good summary of Bitcoin, blockchain, security, network, and privacy. Empirical studies on legal issues surrounding CC include the studies of Böhme et al. (2015), Yermack (2015), Ju et al. (2016), and Lim et al. (2016). Other studies are related to social media (Mai et al. 2018; Xie et al. 2020), investment (Wu and Pandey 2014; Brière et al. 2015; Callen-Naviglia and Alabdan 2016), markets (Bhattacharjee 2016; Malladi and Dheeriya 2021), and currency (Davidson and Block 2015; McCallum 2015; Carrick 2016; Polasik et al. 2016; Li and Wang 2017).

There are many reasons why businesses might want to consider accepting and remitting with bitcoin, and why a firm might hold bitcoin as part of its monetary portfolio. The advantages of using Bitcoin include lower transaction costs (Bunjaku et al. 2017; Gajardo et al. 2018), no inflation risk (Dumitrescu 2017), no boundaries or exchange risk (Bunjaku et al. 2017), faster transactions (Bunjaku et al. 2017), and easy accounting (Tarasova et al. 2020). However, there are potential limitations that firms need to consider. For instance, CCs are very volatile and do not behave like traditional currencies (Gajardo et al. 2018); their accounting rules are not normalized (Tarasova et al. 2020), and they cannot be easily exchanged for paper currencies (Bondarenko et al. 2019). Despite the potential disadvantages, major multinational companies, such as Microsoft, PayPal, Overstock, Wholefoods, and Starbucks have started accepting bitcoin. Additionally, firms such as Tesla, Square, and MicroStrategy are taking positions on CC. This suggests that there is an opportunity, and firms must strategically decide whether they are going to be early movers, fast followers, or late adopters. To make sound use of CCs, a firm has to understand how CCs behave in terms of its other investments and holdings and how it will manage its accounting. For example, choices need to be made about either maintaining their own ledger, using a cloud ledger service, such as Amazon Web Services, or a mix. Hence, we seek to better understand how major CCs behave amidst a global crisis, economic downturn, and supply chain disruptions to contribute to the literature stream and to fill a gap in the literature.

Some research has found that the prices of CC react to variations of both market conditions and economic fundamentals in the short run (Li and Wang 2017), but there is more volatility to the economic fundamentals than to market conditions. From the perspective of an investor, researchers have found that even a modest inclusion of bitcoin in a portfolio improves the risk-return trade-off (Brière et al. 2015).

With the network externality theory, researchers have found that the price of CC is dependent on the following factors: popularity, media sentiment, and trading volume (Polasik et al. 2016; Mai et al. 2018; Xie et al. 2020). Additionally, research suggests that network complexity and flow impact the return and volatility of CCs (Yang and Kim 2015). Yang and Kim (2015) found that as the volume of CC passing through the network increases, forecasts of the return and volatility improve.

Carrick (2016) has questioned whether CCs are currencies in the true sense and whether they meet three criteria of currency. The three criteria used to evaluate whether a token is a currency are, first, it must have the ability to be used as a unit of exchange; second, it must be able to be used as a unit of account; third, it must have an agreed-upon value. Research suggests that bitcoin, at least, seems to act as a currency (Carrick 2016). Furthermore, the author found that portfolios that contain CC outperform those without, and it is a good complement to foreign currency investment. In another study, the findings suggest that bitcoin behaves like gold and the US Dollar (Dyhrberg 2016a). The results of these studies suggest that from a management perspective, CCs can be treated as a currency as they can be used as a unit of exchange and a unit of account, have value, and seem to complement portfolios.

Although CCs may have value, firms want to be certain that concerns over CCs are addressed. First, there is the “double spend” concern, which means that the same digital currency may be spent more than once before the ledger is updated. Second, there is a firm’s concern around transaction costs. Third, there is the potential for fraud. Finally, firms are concerned that parties will not value digital currency. Regarding bitcoin, research has found that the technology that it uses resolves each of the four concerns (Yin et al. 2019). Alzstyne (2014) explained that bitcoin has value because of the following four reasons: (1) the technical aspect of Bitcoin solves the “double spend” problem; (2) transactions are near frictionless; (3) fraud can be detected because of the distributed ledger; and, (4) people value it. However, Bhattacharjee (2016) found that although bitcoin serves as a unit of exchange and account, it fails to act as a store of value. Kristoufek (2015) also found that bitcoin exhibits properties of both traditional financial assets and speculative ones. Given that the technology that digital currencies are based on is relatively new, it is not surprising that the results are inconclusive. One must also acknowledge that some CCs may have been “destroyed” if their owners had forgotten the password to their digital wallets, or the device holding the CC gets “wiped” or destroyed for whatever reason. This problem is also present in non-digital currencies, which may get stolen or destroyed in real life. However, the findings of these studies suggest that, at least, the major CCs behave like currencies. However, given the overall volatility in the value of CC, firms need to be cautious and understand the nature of the volatility.

Some recent studies have found that some of the volatility of CCs can be accounted for by market sentiment and memory (Cheah and Fry 2015; Kristoufek 2015; Malladi and Dheeriya 2021). In these cases, the “memory” of shocks of CC prices is a semi-important determinant of CC prices. However, bitcoin may serve as a good instrument that risk-averse investors can use to protect against negative shocks to the market (Dyhrberg 2016a) and hedge against market-specific risk (Dyhrberg 2016b). A recent study by Grima et al. (2021) suggested that new COVID-19 cases and deaths have had an impact on volatility in major stock markets, especially on the CBOE Volatility Index (VIX).

Not surprisingly, due to the increased investments in CC, researchers were quick to study the potential impact that the COVID-19 pandemic has had on CC. For instance, Umar and Gubareva (2020) applied a wavelet analysis to study the impact of the COVID-19 pandemic on the volatility of the major CCs from January to May of 2020. Their findings suggest that cross-currency hedge strategies are likely to underperform during a global crisis. Comparing it to gold, Kristoufek (2015) examined bitcoin as a safe haven during the pandemic, using the S&P 500 and VIX as benchmarks. The findings of this study reveal that the claims of bitcoin as a safe haven do not hold up during a global crisis. Furthermore, Yousaf and Ali (2021) explored volatility spillovers between the S&P 500 and Litecoin (LTC), Bitcoin, and ETH before and during the pandemic. Their findings suggest that there were no significant spillovers before the pandemic, but during the pandemic, there were unidirectional effects for all the three currencies. Finally, Sahoo (2021) studied the impact of COVID-19 on five of the major CCs from March 2020 to June 2020 using a Granger causality model. The findings suggest a unidirectional causality from the pandemic to the returns.

Although these studies are important to the field of CC, they lack several important features. First, the periods studied in each of the papers only cover the early part of the pandemic. Hence, the studies only captured the initial response of the market, that is, investments in CC, to the pandemic. Second, none of the studies considered the world as a whole or regional reactions to the spread of COVID-19. Finally, none of these studies accounted for a major portion of the CC market.

As noted by Li et al. (2021), the complexity of human behaviors and the changing social environment make it difficult to understand the distribution of financial data. In this study, we extend the existing literature by adding the influence of fear and uncertainty by introducing a pandemic-related period to the evaluation. This is particularly important because, as Aharon et al. (2021) noted, CCs, such as bitcoin, are not independent of other currencies during market stress. In the current study, we use the effective reproductive rate (Spread), which is an epidemiological indicator of the spread of COVID-19, to conduct the analysis. If, as Sahoo (2020) suggests, there is a convergence in the CC market toward clubs, we expect to find similar responses across the major currencies to the spread of the pandemic. Similarly, if, in general, CCs are close to near stock behavior, as found by Sahoo et al. (2019), we expect that CCs perform much like the rest of the market during crises.

Instead of focusing solely on bitcoin, as many studies do, we extend the currencies studied and analyze the price return and trading volume variation of eight of the largest CCs by market capitalization. One of the innovations of the current study is the analysis of an index, defined as the “price return strength,” which is useful in technical analysis because it reveals whether the variation of the price is accompanied by an increase in the trading volume. The "price return strength" is an indicator that reflects the return and the trading volume variation. The market or asset price movements reveal when they are accompanied by a high trading volume. We introduce an indicator that incorporates the direction—first, the return direction and, second, the volume direction. As CCs are traded across the world, we use the effective reproductive variation (or COVID-19 spread) of different geographic areas—Europe, Asia, North America, and the world as a whole.

In this section, we present a summary of the literature and research on CC. We start by specifying some of the advantages and disadvantages of the use of CC by firms as a means of payment for goods and services. We acknowledge that there are some uncertainties about the nature of CC as currency and present some studies that suggest that it does meet the requirements to be considered as a *currency*. However, given the nascent nature of CC, we present some studies that have attempted to account for the volatility in price. In the following section, we present our sample and data. We then discuss the methodological approach employed, followed by the results and discussion.

### Sample and data

The data for the CCs studied are selected based on market capitalization and the availability of data. We select eight digital currencies—Bitcoin, ETH, Tether (USDT), Ripple (XRP), LTC, Bitcoin Cash (BCH), Cardano (ADA), and Binance (BNB). The eight CCs account for nearly 80% of the total market capitalization of all CCs. The period analyzed is from March 1, 2020 to December 15, 2021 (450 observations), which is roughly during the period of the pandemic to the time of submission of this paper. To determine the effect of COVID-19 on CCs, we use the spread as the key variable. As CCs are global currencies, we analyze the impact of the daily variation of the effective reproductive rate (Spread) worldwide ($r_{{covid_{{world_{t} }} }}$) as well as in North America ($r_{{covid_{{north\;america_{t} }} }}$), Europe ($r_{{covid_{{europe_{t} }} }}$), and Asia ($r_{{covid_{{asia_{t} }} }}$). The data about new COVID-19 cases are extracted from Our World in Data of the Oxford Martin School, University of Oxford. The Spread is computed from January 26, 2020 onwards. To calculate the Spread, we use the methodology proposed by Cori et al. (2013).

The $r_{{covid_{t} }}$ index is based on two definitions. First, we let *w*_*s*_ be the probability distribution of infection; because *s* is more infectious when *w*_*s*_ is greater, it does not directly depend on calendar time. Second, we let *I*_*t*_ be the number of new infections at time *t*; i.e., the reported daily cases. Thus, $r_{{covid_{t} }}$ can be calculated as the ratio of the expected new cases in time *t* to the total infected individuals at time *t*. Mathematically, we derive the following:

1 $$r_{{covid_{t} }} = \frac{{E\left[ {I_{t} } \right]}}{{\mathop \sum \nolimits_{s = 1}^{t} I_{t - s} w_{s} }}$$

We use the “EpiEstim” in the R package to calculate *R*_*t*_ created by Cori et al. (2013). We use this approach because it is very easy to employ it in non-epidemiological research but manages to preserve the robustness of previous approaches of real-time monitoring of a pandemic. Additionally, it has become the standard index to monitor how a country or lower-level administrations are handling COVID-19.

In this study, we analyze three main variables of the CCs. The first variable is the daily variation in the price of CC ($r_{{crypto_{t} }}$); the second is the variation in the daily trading volume ($v_{{crypto_{t} }}$); third is the return strength of the price of CC ($s_{{crypto_{t} }}$). The last indicator is defined as the absolute value of the CC daily price return multiplied by the variation in the daily trading volume. This index reveals whether the changes in the return are supported by higher or lower trading volume to determine the intensity of the movement.

**Fig. 1:**
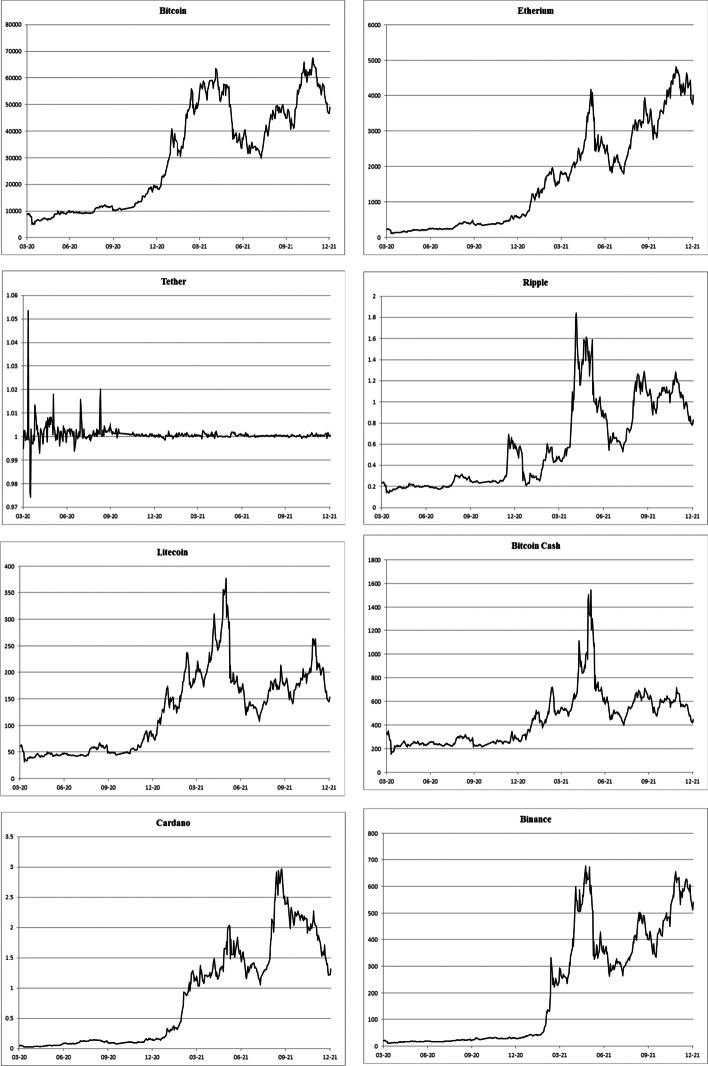
Price Evolution of the main CCs

As illustrated in Fig. 1, all the CCs had a price peak in May 2021, except for USDT. USDT saw a peak at the beginning of the pandemic in March 2020, followed by a period of volatility and then a fairly stable price from September 2020. For the other CCs, after this spike in May 2020, each of the price series dropped, and XRP, LTC, and BCH failed to regain their high growth rate, whereas the other regained or surpassed the May 2021 high. In the case of bitcoin, from March 2020, the price started to rally, increasing the upward trend in the second half of 2020, followed by a drop in June 2021 and rebounding by the end of the year. The price of ETH began a rally from the beginning of 2020—around the start of the COVID-19 pandemic. Finally, BNB had an extremely large gain in March 2020, and it fell and recovered its near high level at the end of 2021.

Due to the existence of heteroscedasticity in most financial time series, Engle (1982) proposed the Autoregressive Conditional Heteroskedasticity model (ARCH). Bollerslev (1986) generalized the ARCH model with the Generalized Autoregressive Conditional Heteroskedasticity (GARCH) model to address the time series volatility that changes over time.

### Models

**Fig. 2:**
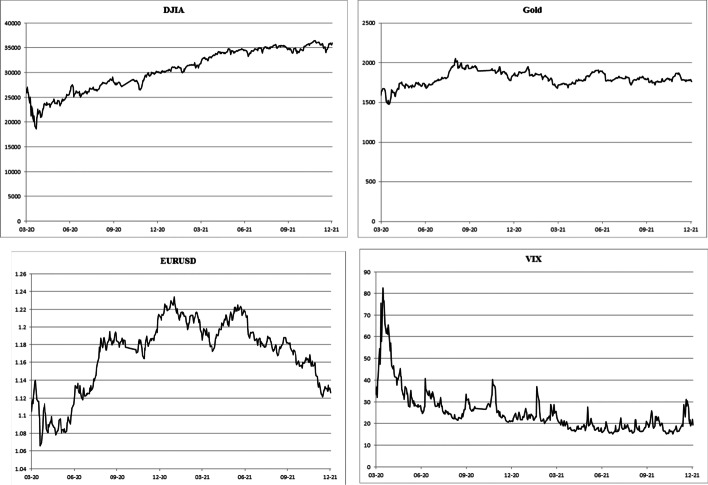
Price evolution of the DJIA, GOLD, EURUSD, and VIX during the COVID-19 period

To effectively determine the impact of COVID-19 spread on CCs and taking into account the time-varying property of volatilities, the GARCH model (Bollerslev 1986) is applied using control variables and an autoregressive model with lag *k* in the returns. The time series of the Dow Jones Industrial Average (DJIA), Gold price (GOLD), EURO–US Dollar rate (EURUSD), and VIX are employed as control variables. In Fig. 2, the evolution of the control variables is presented. Each series had a different behavior during the COVID-19 period. For instance, as illustrated in Fig. 2, whereas DJIA had a sharp decline at the beginning of the pandemic, it had a steady climb from around April 2020 to December 2021. During the same period, gold was largely flat; the Euro to US Dollar dropped, rose, and then declined again; finally, the VIX had a sharp but brief rise in March 2020, followed by a greater decline that it never recovered from.

For each of the three main characteristics series of CC, two models are defined—one for the worldwide COVID-19 effect and the other for the North American, European, and Asian COVID-19 effects. To fit the models, the Augmented Dickey–Fuller (ADF) test is conducted to check the stationarity of the time series.

The first two models related to the CC price variation are described in Eq. 2 for Worldwide COVID-19 effects and in Eq. 3 for North American, European, and Asian COVID-19 effects. In each equation, for each CC, the lags *k, m, n, o, p, q*, and *w* are adjusted based on the minimized Akaike Information Criterion (AIC). The maximum for *k* is 5 days*, for l, m, n, o, p*, and *q* is 2 days; the absolute threshold *w* is 1.

2a $$r_{{crypto_{t} }} = \alpha_{0} + \mathop \sum \limits_{i = 1}^{k} \beta_{i} r_{{crypto_{t - i} }} + \gamma_{w} r_{{{\text{cov}} id_{{world_{t} }} }} + \mathop \sum \limits_{i = 1}^{l} \delta_{i} r_{{dji_{t - i} }} + \mathop \sum \limits_{i = 1}^{m} \theta_{i} r_{{gold_{t - i} }} + \mathop \sum \limits_{i = 1}^{n} \vartheta_{i} r_{{eur_{t - i} }} + \mathop \sum \limits_{i = 1}^{o} \pi_{i} r_{{vix_{t - i} }} + e_{t}$$

2b $$e_{t} \sim N\left( {0,\sigma_{t}^{2} } \right)$$

2c $$\sigma_{t}^{2} = \mu + \mathop \sum \limits_{i = 1}^{p} \tau_{i} \sigma_{t - i}^{2} + \mathop \sum \limits_{i = 1}^{w} \kappa_{i} \left| {e_{t - i} } \right|^{2} + \mathop \sum \limits_{i = 1}^{q} \omega_{i} e_{t - i}^{{2}{}}$$

3a $$\begin{aligned} & r_{{crypto_{t} }} = \alpha_{0} + \mathop \sum \limits_{i = 1}^{k} \beta_{i} r_{{crypto_{t - i} }} + \gamma_{na} r_{{covid_{{america_{t} }} }} + \gamma_{e} r_{{covid_{{europe_{t} }} }} + \gamma_{a} r_{{covid_{{asia_{i} }} }} \\ & \quad + \mathop \sum \limits_{i = 1}^{l} \delta_{i} r_{{dji_{t - i} }} + \mathop \sum \limits_{i = 1}^{m} \theta_{i} r_{{gold_{t - i} }} + \mathop \sum \limits_{i = 1}^{n} \vartheta_{i} r_{{eur_{t - i} }} + \mathop \sum \limits_{i = 1}^{o} \pi_{i} r_{vix} + e_{t} \\ \end{aligned}$$

3b $$e_{t} \sim N\left( {0,\sigma_{t}^{2} } \right)$$

3c $$\sigma_{t}^{2} = \mu + \mathop \sum \limits_{i = 1}^{p} \tau_{i} \sigma_{t - i}^{2} + \mathop \sum \limits_{i = 1}^{w} \kappa_{i} \left| {e_{t - i} } \right|^{2} + \mathop \sum \limits_{i = 1}^{q} \omega_{i} e_{t - i}^{2}$$

To analyze the second variable of CC, that is, the daily variation in trading volume ($$v_{{crypto_{t} }}$$), we propose 2 models for COVID-19 cases worldwide and in North America, Europe, and Asia. The first model is similar to the return models described in Eqs. 1 and 2, but the only change is that the dependent variable and the autoregressive variable are $$v_{{crypto_{t} }}$$ instead of $$r_{{crypto_{t} }}$$. The second model is based on the volume change. A large jump in new cases or a sharp decrease in new cases could cause a shock that increases the trading volume. The second model adds the variation in the daily Spread in absolute value ($$rav_{{covid_{t} }} )$$, as described in Eqs. 4 and 5.

4a $$\begin{aligned} & v_{{crypto_{t} }} = \alpha _{0} + \mathop \sum \limits_{{i = 1}}^{k} \beta _{i} v_{{crypto_{{t - i}} }} + \gamma _{w}^{\prime } rav_{{covid_{{world_{t} }} }} \\ & \quad \quad \quad \quad + \mathop \sum \limits_{{i = 1}}^{l} \delta _{i} r_{{dji_{{t - i}} }} + \mathop \sum \limits_{{i = 1}}^{m} \theta _{i} r_{{gold_{{t - i}} }} + \mathop \sum \limits_{{i = 1}}^{n} \vartheta _{i} r_{{eur_{{t - i}} }} + \mathop \sum \limits_{{i = 1}}^{o} \pi _{i} r_{{vix_{{t - i}} }} + e_{t} \\ \end{aligned}$$

4b $$e_{t} \sim N\left( {0,\sigma_{t}^{2} } \right)$$

4c $$\sigma_{t}^{2} = \mu + \mathop \sum \limits_{i = 1}^{p} \tau_{i} \sigma_{t - i}^{2} + \mathop \sum \limits_{i = 1}^{w} \kappa_{i} \left| {e_{t - i} } \right|^{2} + \mathop \sum \limits_{i = 1}^{q} \omega_{i} e_{t - i}^{2}$$

5a $$\begin{aligned} & v_{{crypto_{t} }} = \alpha_{0} + \mathop \sum \limits_{i = 1}^{k} \beta_{i} v_{{crypto_{t - i} }} + \gamma_{na}^{\prime } rav_{{covid_{{america_{t} }} }} + \gamma_{e}^{\prime } rav_{{covid_{{europe_{t} }} }} + \gamma_{a}^{\prime } rav_{{covid_{{asia_{i} }} }} \\ & \quad \quad \quad \quad + \mathop \sum \limits_{i = 1}^{l} \delta_{i} r_{{dji_{t - i} }} + \mathop \sum \limits_{i = 1}^{m} \theta_{i} r_{{gold_{t - i} }} + \mathop \sum \limits_{i = 1}^{n} \vartheta_{i} r_{{eur_{t - i} }} + \mathop \sum \limits_{i = 1}^{o} \pi_{i} r_{{vix_{t - i} }} + e_{t} \\ \end{aligned}$$

5b $$e_{t} \sim N\left( {0,\sigma_{t}^{2} } \right)$$

5c $$\sigma_{t}^{2} = \mu + \mathop \sum \limits_{i = 1}^{p} \tau_{i} \sigma_{t - i}^{2} + \mathop \sum \limits_{i = 1}^{w} \kappa_{i} \left| {e_{t - i} } \right|^{2} + \mathop \sum \limits_{i = 1}^{q} \omega_{i} e_{t - i}^{2}$$

Finally, we consider the price return strength of CC ($s_{{crypto_{t} }}$). This indicator reveals that the price changes are relevant when they are accompanied by higher trading volume. Then, the price return strength is calculated as the product of the daily return and the absolute value of the variation in daily volume. This indicator captures the price movements or trend generation better. We analyze two models—the variation of the daily Spread (Eqs. 5 and 6) and replacing the variation of the daily Spread with their absolute value ($rav_{{covid_{t} }}$ instead of $r_{{covid_{t} }}$ in Eqs. 6 and 7).

6a $$s_{{crypto_{t} }} = \,\alpha_{0} + \mathop \sum \limits_{i = 1}^{k} \beta_{i} s_{{crypto_{t - i} }} + \gamma_{w} r_{{covid_{{world_{t} }} }} + \mathop \sum \limits_{i = 1}^{l} \delta_{i} r_{{dji_{t - i} }} + \mathop \sum \limits_{i = 1}^{m} \theta_{i} r_{{gold_{t - i} }} + \mathop \sum \limits_{i = 1}^{n} \vartheta_{i} r_{{eur_{t - i} }} + \mathop \sum \limits_{i = 1}^{o} \pi_{i} r_{{vix_{t - i} }} + e_{t}$$

6b $$e_{t} \sim N\left( {0,\sigma_{t}^{2} } \right)$$

6c $$\sigma_{t}^{2} = \mu + \mathop \sum \limits_{i = 1}^{p} \tau_{i} \sigma_{t - i}^{2} + \mathop \sum \limits_{i = 1}^{w} \kappa_{i} \left| {e_{t - i} } \right|^{2} + \mathop \sum \limits_{i = 1}^{q} \omega_{i} e_{t - i}^{2}$$

7a $$\begin{aligned} & s_{{crypto_{t} }} = \alpha _{0} + \mathop \sum \limits_{{i = 1}}^{k} \beta _{i} s_{{crypto_{{t - i}} }} + \gamma _{{na}} r_{{covid_{{america_{t} }} }} + ~\gamma _{e} r_{{covid_{{europe_{t} }} }} + ~\gamma _{a} r_{{covid_{{asia_{i} }} }} ~ \\ & \quad \quad \quad \quad + \mathop \sum \limits_{{i = 1}}^{l} \delta _{i} r_{{dji_{{t - i}} }} + \mathop \sum \limits_{{i = 1}}^{m} \theta _{i} r_{{gold_{{t - i}} }} + \mathop \sum \limits_{{i = 1}}^{n} \vartheta _{i} r_{{eur_{{t - i}} }} + \mathop \sum \limits_{{i = 1}}^{o} \pi _{i} r_{{vix_{{t - i}} }} + e_{t} \\ \end{aligned}$$

7b $$e_{t} \sim N\left( {0,\sigma_{t}^{2} } \right)$$

7c $$\sigma_{t}^{2} = \mu + \mathop \sum \limits_{i = 1}^{p} \tau_{i} \sigma_{t - i}^{2} + \mathop \sum \limits_{i = 1}^{w} \kappa_{i} \left| {e_{t - i} } \right|^{2} + \mathop \sum \limits_{i = 1}^{q} \omega_{i} e_{t - i}^{2}$$

## Results

The ADF test is used to test for the stationarity of all time series studied. In all our time series, the ADF test is rejected, indicating the stationarity of the series (Table 6).

The hypothesis for Model 1 is that an increase in the Spread negatively influences the price of CC. The results demonstrate that the Spread variation of the world as a whole does not affect the price return of the major CCs, except for USDT (See Table 1, Model 1A). The results of Model 1B reveal that the European Spread variation does not have a significant impact; the North American Spread variation has a negative effect on USDT, and the Asian Spread variation has a negative effect on BNB, whereas it has a positive effect on XRP and BCH.

**Table 1:** CC return price models are explained by the variation in the effective reproductive rate

	COVID World		COVID North America		COVID Europe		COVID Asia	
Crypto	Coef	*p*-value	Coef	*p*-value	Coef	*p*-value	Coef	*p*-value
BTC	0.0050	0.8756	0.0123	0.7551	− 0.0134	0.6931	− 0.0227	0.4125
ETH	− 0.0428	0.1450	0.0234	0.6519	− 0.0857	0.0646	− 0.0227	0.4777
USDT	− **0.0009**	**0.0013**	− **0.0016**	**0.0001**	− 0.0007	0.1679	− 0.0002	0.6771
XRP	0.0291	0.2038	0.0426	0.1936	− 0.0100	0.8094	**0.0405**	**0.0428**
LTC	− 0.0294	0.5171	0.0483	0.3216	− 0.0220	0.6774	− 0.0579	0.1168
BCH	0.0014	0.9821	− 0.0498	0.2610	0.0011	0.9836	**0.0656**	**0.0021**
ADA	− 0.0292	0.5641	0.0009	0.9876	− 0.1008	0.1084	− 0.0428	0.3679
BNB	− 0.0205	0.4438	− 0.0207	0.7234	0.0516	0.2878	− **0.0498**	**0.0402**

The control variables used for Eq. 2 (COVID-19 cases worldwide) and Eq. 3 (COVID cases in North America, Europe, and Asia) are CC price return autoregressive terms, DJIA returns lagged, GOLD variations lagged, EURUSD variations lagged, and VIX variations lagged. The bold numbers are coefficients significant at the 5% level. All other parameters of the models are presented in Tables 7 and 8

The hypothesis for Model 2 is that an increase in the Spread negatively influences the trading volume of CC. This hypothesis is mainly supported if a decrease in the Spread positively impacts the trading volume. The worldwide and North American Spreads positively impact BCH’s trading volume, and the Asian Spread has a negative relationship with XRP’s trading volume (See Table 2).

**Table 2:** CC daily trading volume variations models are explained by the variation in the effective reproductive rate

	COVID World		COVID North America		COVID Europe		COVID Asia	
Crypto	Coef	*p*-value	Coef	*p*-value	Coef	*p*-value	Coef	*p*-value
BTC	0.0391	0.5906	− 0.0364	0.8426	0.1644	0.4363	0.0000	0.9997
ETH	− 0.0210	0.7780	− 0.1250	0.4791	0.1281	0.4919	− 0.0447	0.7064
USDT	0.1314	0.2610	− 0.2132	0.2008	0.3209	0.1035	0.1190	0.3707
XRP	0.1307	0.3244	− 0.0749	0.7629	0.2500	0.3513	**0.1691**	**0.0006**
LTC	− 0.0454	0.6804	− 0.1338	0.4267	− 0.0945	0.6068	− 0.0173	0.8841
BCH	**0.1625**	**0.0461**	**0.7108**	**0.0006**	− 0.0808	0.7944	0.0557	0.5916
ADA	0.1224	0.4437	0.0373	0.9053	0.0116	0.9755	0.0838	0.6057
BNB	0.0273	0.7826	− 0.2095	0.1325	0.1932	0.2955	0.0136	0.8982

The control variables used are CC price return autoregressive terms, DJIA returns lagged, GOLD variations lagged, EURUSD variations lagged, and VIX variations lagged. The bold numbers are coefficients significant at the 5% level. All the other parameters of the models are presented in Tables 9 and 10

However, a large increase in the number of new cases can also lead to an increase in price and trading volume. Therefore, we fit the same models, but with the absolute value of the Spread. The worldwide Spread affects the trading volume of BCH. The absolute value of the variation of the daily spread in North America positively impacts LTC, whereas the absolute value of the variation of the daily Spread in Asia impacts BCH (see Table 3).

**Table 3:** CC variations in daily trading volume models are explained by the absolute variation of the effective reproductive rate

	COVID World		COVID North America		COVID Europe		COVID Asia	
Crypto	Coef	*p*-value	Coef	*p*-value	Coef	*p*-value	Coef	*p*-value
BTC	− 0.0418	0.7051	0.0780	0.7302	0.0266	0.9095	− 0.0055	0.9352
ETH	0.0277	0.8631	0.0641	0.8043	0.4126	0.0961	− 0.0035	0.9798
USDT	0.0052	0.9560	0.0688	0.7302	0.2672	0.2806	0.0759	0.6071
XRP	− 0.0158	0.8627	− 0.1886	0.4849	0.4200	0.1438	− 0.0119	0.8896
LTC	0.0279	0.8261	**0.4040**	**0.0442**	0.0044	0.9846	0.0035	0.9799
BCH	**0.2293**	**0.0036**	0.2558	0.4103	− 0.3908	0.2630	**0.2417**	**0.0047**
ADA	− 0.1382	0.4324	− 0.1760	0.6642	0.5351	0.2819	− 0.1916	0.2716
BNB	0.0541	0.4489	0.3554	0.1576	0.0092	0.9610	0.0179	0.8703

The control variables used include CC price return autoregressive terms, DJIA returns lagged, GOLD variations lagged, the EURUSD variations lagged, and VIX variations lagged. The bold numbers are coefficients significant at the 5% level. All other parameters of the models are presented in Tables 11 and 12

Finally, the price return strength of CC is analyzed. The first model reveals the reaction direction of the price of CC to the Spread variation (Table 4). The reaction of the prices varies across all CCs analyzed. The results imply that when the Spread decreases (increases), it leads to an increase (decrease) in the price of CC, which is accompanied by an increase in trading volume. This finding demonstrates the relevance and impact of COVID-19 cases on the price of CCs. In particular, the worldwide Spread influenced the prices of BCH and USDT. The European Spread affected ETH, BCH, and XRP. Regarding XRP, BNB, ETH, and BCH, they were also affected by the North American Spread, whereas the Asian Spread impacted ETH, USDT, BCH, BNB, and XRP.

**Table 4:** CC price return strength models are explained by the variation in the effective reproductive rate

	COVID World		COVID North America		COVID Europe		COVID Asia	
Crypto	Coef	*p*-value	Coef	*p*-value	Coef	*p*-value	Coef	*p*-value
BTC	0.0049	0.3629	− 0.0067	0.2751	0.0151	0.1215	0.0055	0.3292
ETH	− **0.0082**	**0.0110**	− **0.0195**	**0.0206**	**0.0502**	**0.0000**	− **0.0090**	**0.0190**
USDT	− **0.0003**	**0.0001**	− 0.0001	0.3389	− 0.0001	0.3046	− **0.0006**	**0.0000**
XRP	0.0120	0.4094	**0.0661**	**0.0000**	− **0.0559**	**0.0005**	− **0.0205**	**0.0000**
LTC	0.0089	0.5401	0.0079	0.5189	0.0148	0.1583	− 0.0072	0.6665
BCH	**0.0249**	**0.0000**	**0.0154**	**0.0051**	− **0.0276**	**0.0002**	**0.0277**	**0.0000**
ADA	0.0135	0.6710	0.0150	0.5561	0.0110	0.7534	0.0106	0.6879
BNB	0.0036	0.6058	− **0.0419**	**0.0000**	0.0173	0.1845	**0.0146**	**0.0341**

The control variables used include CC price return autoregressive terms, DJIA returns lagged, GOLD variations lagged, EURUSD variations lagged, and VIX variations lagged. The bold numbers represent coefficients significant at the 5% level. All other parameters of the models are presented in Tables 13 and 14

In the final model, we use the absolute value of variations of the Spread to measure the price of CC independently. The worldwide results reveal a positive effect on ETH and BCH and negative effects on USDT, ADA, and BNB, indicating that larger variations of the Spread led to an increase in the price of ETH and BCH and a decrease in the price of USDT, ADA, and BNB. However, for the analysis by region, the Asian Spread variation had a positive effect, which is contrary to the result of the worldwide model USDT. In the Asian markets, the results reveal negative effects on LTC, ANA, and BNB, whereas there are positive effects on USDT and BCH. The variation of the North American Spread had positive effects on Bitcoin and ADA, whereas it had negative effects on ETH, XRP, and LTC. In Europe, the variation of the spread had significant positive effects on the price of ETH and USDT, whereas it had negative effects on LTC. Hence, this model does not demonstrate a clear effect of the size of the variation of Spread (Table 5).

**Table 5:** CCs price return strength models are explained by the absolute value of the variation in effective reproductive rate

	COVID World		COVID North America		COVID Europe		COVID Asia	
Crypto	Coef	*p*-value	Coef	*p*-value	Coef	*p*-value	Coef	*p*-value
BTC	− 0.0049	0.3477	**0.0181**	**0.0425**	− 0.0102	0.4087	− 0.0047	0.3974
ETH	**0.0060**	**0.0259**	− **0.0427**	**0.0006**	**0.1440**	**0.0000**	0.0054	0.3044
USDT	− **0.0002**	**0.0013**	0.0000	0.8187	**0.0008**	**0.0000**	**0.0007**	**0.0000**
XRP	− 0.0080	0.5695	− **0.0640**	**0.0088**	− 0.0117	0.6544	− 0.0191	0.2149
LTC	0.0004	0.9763	− **0.0387**	**0.0033**	− **0.0266**	**0.0377**	− **0.0242**	**0.0034**
BCH	**0.0226**	**0.0000**	− 0.0142	0.0858	0.0056	0.5967	**0.0254**	**0.0000**
ADA	− **0.0414**	**0.0403**	**0.0714**	**0.0000**	− 0.0182	0.3069	− **0.0629**	**0.0000**
BNB	− **0.0236**	**0.0000**	0.0178	0.2156	− 0.0108	0.3433	− **0.0329**	**0.0000**

The control variables used in Eq. 5 (COVID cases worldwide) and Eq. 6 (COVID-19 cases in North America, Europe, and Asia) are CCs price returns autoregressive terms, DJIA returns lagged, GOLD variations lagged, EURUSD variations lagged, and VIX variations lagged, according. In bold are the coefficients significant at the 5% level. All the other parameters of the models are presented in Tables 15 and 16

## Discussion

As suggested in the literature review, researchers have studied the impact that COVID-19 has had on CC. Umar and Gubareva (2020) conducted a time–frequency analysis of the volatility of CC markets, which is induced by the pandemic. Umar et al. (2021a, b) studied the impact of media coverage of the pandemic on the return and volatility of CCs. Sahoo (2021) explored the linear and nonlinear causal relationship between COVID-19 and CC markets. Yousaf and Ali (2021) analyzed the relationships between stock and CC markets. Moreover, Kristoufek (2020) assessed bitcoin as a safe haven during the pandemic. Each of these studies has helped improve our understanding of the CC market, but these studies were conducted at the early stages of the pandemic, mostly focusing on the first quarter. Unlike these earlier studies, this research considers almost the two years that the pandemic has persisted.

Furthermore, the earlier studies reviewed did not consider the magnitude of the pandemic but rather considered the relationship between the market and the pandemic (e.g., Kristoufek 2020; Yousaf and Ali 2021), the relationship between media and CCs (e.g., Umar et al. 2021a, b), or the time series (Umar and Gubareva 2020). The current study expands our knowledge of the literature by including an indicator for the spread of the pandemic. We present several models in which we analyze the impact that the spread of COVID-19 has on the price of eight major CCs. The spread is an epidemiological indicator that shows whether an epidemic is under control or not.

Previous research in this area also lacks any assessment of the global and/or regional impacts that the pandemic has had on the value of CC. The current study further expands our understanding of CC markets by looking at the impact of the pandemic on global and regional markets for CC. We analyze the aforementioned effect at the global level because CC is traded globally. We also analyze the effect at the regional level because the impact is likely not going to be uniform. We hypothesize that as the spread of the virus increases, the price of CC would decrease. As stated in the results section, the effect of the spread of COVID-19 on the price of CC is not uniform, either by currency or region. We find statistically significant impacts but the fact that they are not uniform suggests that there is still more work to be done in this stream of research. Although they are not tested in this study, there are various potential reasons for the lack of uniformity. First, the predominance of the use of a particular CC may vary by region and, hence, have greater importance. Second, it may be that the impacts of various currencies in some regions are greater (lesser) because people change their use of the currency during the waxing and waning of the pandemic. Third, as suggested in the introduction to the discussion of the nature of CC, it may be that some of the major currencies behave like stocks, and there may be an impact by “clubs,” as suggested by a Sahoo (2020). These potential rationales point to areas for future studies. The lessons learned from these potential areas for future studies can help firms define protocols for the implementation and use of CCs during crises.

An additional area of innovation presented in this study is the inclusion of an analysis of an index that we defined as price return strength. This index is useful for technical analysis because it reveals whether the variation of the CC price is accompanied by an increase in the trading volume. As mentioned in the results section, all the CCs analyzed had a negative reaction, except BNB. The implication is that when the price of the CC goes up as a result of a decrease in the Spread, the trading volume also goes up. There are various potential explanations for such behavior. For instance, one might assume that people take advantage of the gains. Although it is not tested in this study, this could be an interesting area for future research.

Although it is not considered in this study, there is an opportunity to conduct further analysis into this. We expect that although there may not be significant results when aggregated, there may be significant results at the country level. For instance, we find that for Asia, only BNB is impacted significantly. If we were to look at specific countries in Asia, we may find different results. However, it may be that in aggregate, BNB is the most widely adopted CC in Asia; therefore, it is more significantly impacted. A measure of the percentage of trading by country may be one way to address this issue.

One can extend this study to other asset classes, such as equities and bonds. It would be interesting to determine whether the effect of the COVID-19 Spread on major stock indices is similar to that of gold and other commodity prices. The impact of the COVID-19 spread on equity prices may be more significant because the trading volume and market capitalization of equities are far greater than that of CCs. Such an analysis can be another extension of this study.

## Conclusions

In this study, we extend the body of knowledge on CC, but there is still a lot of research to be done in this area before it becomes generally accepted. Nonetheless, there are still many opportunities for firms to take advantage of temporary market inefficiencies if approached strategically. Early adopters of CC may be able to capture a segment of the market that has and wants to use CC before their competitors gain the ability to do so.

Although most organizations may not be structurally capable to take advantage of the nascent nature of CC, there is an opportunity for those that can develop competencies that allow them to take full advantage of the early learning effects. For instance, locating the currency in havens that do not tax CCs as income but rather as an asset is one strategy. Another approach firms can adopt is to integrate the transactions into other blockchain activities to strategically develop complete digital ledgers and smart contracts.

Finally, we recognize that an early entry into CC is not something to be undertaken lightly. Firms that enter into this domain ought to have the capacity to research and monitor their CC assets. Failure to manage the asset will likely result in loss, as would failure to manage any other corporate asset.

## Data Availability

The data used in this research paper are public, obtained from the CoinMarketCap site (https://coinmarketcap.com/) and from the Coin Metrics site (https://coinmetrics.io/).

## Appendix

See Tables 6, 7, 8, 9, 10, 11, 12, 13, 14, 15, 16.

**Table 6:** Augmented Dickey–Fuller TEST

Cryptocurrency	ADF	*p*-value	Stock Market or Commodity	ADF	*p*-value
**Price Return Series**			**Price Return Series**		
Bitcoin USD	− 23,9097	0,0000	DJI	− 7,6237	0,0000
Ethereum USD	− 25,1079	0,0000	GOLD	− 21,6440	0,0000
Tether USD	− 12,6275	0,0000	EUR	− 20,6818	0,0000
XRP USD	− 22,4269	0,0000	VIX	− 25,6374	0,0000
Litecoin USD	− 24,5623	0,0000			
BitcoinCash USD	− 24,7690	0,0000			
Cardano USD	− 23,8509	0,0000			
BinanceCoin USD	− 22,8648	0,0000			

Cryptocurrency	ADF	*p*-value	Geographic Area	ADF	*p*-value
**Volume Variation Series**			**Spread (Effective Reproductive Var.) Series**		
Bitcoin USD	− 19,5472	0,0000	Asia	− 11,2878	0,0000
Ethereum USD	− 19,4058	0,0000	Europe	− 10,2362	0,0000
Tether USD	− 18,2420	0,0000	North America	− 11,1357	0,0000
XRP USD	− 17,8793	0,0000	World	− 10,8714	0,0000
Litecoin USD	− 16,3889	0,0000			
BitcoinCash USD	− 16,1807	0,0000			
Cardano USD	− 17,3640	0,0000			
BinanceCoin USD	− 17,7069	0,0000			
**Price Return Strength Series**			**Spread (Eff. Rep. Abs. Var.) Series**		
Bitcoin USD	− 24,2445	0,0000	Asia	− 3,7795	0,0034
Ethereum USD	− 17,9780	0,0000	Europe	− 4,9803	0,0000
Tether USD	− 7,6231	0,0000	North America	− 4,7104	0,0001
XRP USD	− 17,8657	0,0000	World	− 3,7480	0,0037
Litecoin USD	− 18,2731	0,0000			
BitcoinCash USD	− 23,0945	0,0000			
Cardano USD	− 18,2918	0,0000			
BinanceCoin USD	− 26,5780	0,0000	−

**Table 7:** F-test for the rest of coefficients of the Model 1A

	Autoregresive		DJI		Gold		EUR		VIX	
Crypto	F-AR	*p*-value	FDJI-AR	*p*-value	FGOLD-AR	*p*-value	FEUR-AR	*p*-value	FVIX-AR	*p*-value
BTC	1.5725	0.2098	2.8623	0.0581	**3.5635**	**0.0291**	**3.3235**	**0.0196**	**4.1495**	**0.0164**
ETH	0.8150	0.3667	**7.1877**	**0.0073**	0.0004	0.9843	**3.9416**	**0.0201**	0.1032	0.7480
USDT	**35.6107**	**0.0000**	**31.3590**	**0.0000**	**15.6881**	**0.0001**	**17.9479**	**0.0000**	3.6376	0.0565
XRP	**4.5351**	**0.0332**	**8.8316**	**0.0000**	0.1558	0.6930	0.2717	0.6022	**9.7421**	**0.0000**
LTC	1.3763	0.2407	1.5569	0.2121	2.4810	0.0604	**5.9300**	**0.0029**	1.5409	0.2153
BCH	0.4317	0.7857	**3.5909**	**0.0283**	**5.6153**	**0.0039**	3.1829	0.0744	2.3882	0.0929
ADA	0.0413	0.8389	1.2870	0.2771	0.1710	0.6793	1.8571	0.1573	0.3653	0.6942
BNB	0.1992	0.6554	1.9372	0.1640	0.0288	0.8652	**6.3683**	**0.0019**	1.7104	0.1819

In bold the coefficients significant at 5% level

**Table 8:** F-test for the rest of coefficients of the Model 1B

	Autoregresive		DJI		Gold		EUR		VIX	
Crypto	F-AR	*p*-value	FDJI-AR	*p*-value	FGOLD-AR	*p*-value	FEUR-AR	*p*-value	FVIX-AR	*p*-value
BTC	0.1557	0.6931	2.2909	0.1023	**3.5803**	**0.0286**	1.2735	0.2828	**3.7144**	**0.0251**
ETH	3.4158	0.0646	1.0505	0.3054	0.0380	0.8454	**3.6996**	**0.0255**	0.0925	0.7610
USDT	**19.4794**	**0.0000**	**30.6705**	**0.0000**	**17.9272**	**0.0000**	**13.5277**	**0.0000**	**5.9440**	**0.0148**
XRP	0.7987	0.3715	**4.8015**	**0.0026**	0.7360	0.3910	0.3886	0.5330	**7.3755**	**0.0001**
LTC	0.1730	0.6774	**4.2646**	**0.0389**	2.2977	0.1016	**11.2957**	**0.0000**	0.2841	0.5940
BCH	0.0004	0.9836	2.4464	0.1178	**2.8160**	**0.0388**	2.3011	0.1013	1.5936	0.2043
ADA	2.5768	0.1084	0.0703	0.7909	0.0016	0.9685	0.1867	0.6657	0.3961	0.6732
BNB	1.3860	0.2391	1.7148	0.1904	0.0933	0.7600	**5.7915**	**0.0033**	1.6697	0.1894

In bold the coefficients significant at 5% level

**Table 9:** F-test for the rest of coefficients of the Model 2A

	Autoregresive		DJI		Gold		EUR		VIX	
Crypto	F-AR	*p*-value	FDJI-AR	*p*-value	FGOLD-AR	*p*-value	FEUR-AR	*p*-value	FVIX-AR	*p*-value
BTC	**21.9832**	**0.0000**	0.3547	0.5514	0.1874	0.8292	0.1015	0.7501	2.1549	0.1421
ETH	**23.6377**	**0.0000**	0.5474	0.5788	1.2657	0.2606	0.3955	0.7563	0.7643	0.3820
USDT	**7,353,091.0000**	**0.0000**	**6.5004**	**0.0003**	0.3691	0.5435	2.1689	0.1408	**10.8215**	**0.0010**
XRP	**15.6709**	**0.0000**	0.0650	0.7988	1.0035	0.3910	0.7177	0.5418	0.8321	0.4358
LTC	**204.3899**	**0.0000**	2.0556	0.1517	0.3967	0.5288	2.0571	0.1515	0.1886	0.6641
BCH	**7.5811**	**0.0006**	1.0822	0.3563	0.1693	0.6807	**3.6691**	**0.0123**	0.6533	0.5208
ADA	**14.1278**	**0.0000**	0.8912	0.3451	1.7806	0.1821	**85.3342**	**0.0000**	2.4361	0.0886
BNB	**15.0211**	**0.0000**	0.4880	0.6142	0.0191	0.8900	0.6015	0.6143	0.8556	0.4641

In bold the coefficients significant at 5% level

**Table 10:** F-test for the rest of coefficients of the Model 2B

	Autoregresive		DJI		Gold		EUR		VIX	
Crypto	F-AR	*p*-value	FDJI-AR	*p*-value	FGOLD-AR	*p*-value	FEUR-AR	*p*-value	FVIX-AR	*p*-value
BTC	**16.9232**	**0.0000**	1.5042	0.2127	0.1102	0.7399	0.4323	0.6493	2.2710	0.1318
ETH	**17.8203**	**0.0000**	0.8553	0.3550	1.4523	0.2282	0.4573	0.4989	0.6763	0.4109
USDT	**18.1483**	**0.0000**	1.3675	0.2558	1.9462	0.1630	0.0303	0.8617	2.6557	0.1032
XRP	**7.1361**	**0.0001**	0.0147	0.9034	1.8943	0.1687	0.2723	0.6018	0.6988	0.5532
LTC	0.1483	0.8622	1.0027	0.3167	0.3239	0.5693	0.8994	0.3430	0.0665	0.7965
BCH	1.3861	0.2511	0.9316	0.4252	0.0683	0.9768	**2.9773**	**0.0313**	0.4738	0.7007
ADA	**7.3338**	**0.0001**	0.6345	0.4257	1.8230	0.1770	**42.9234**	**0.0000**	2.0797	0.1261
BNB	**14.3446**	**0.0000**	0.4935	0.6869	0.0023	0.9620	0.8156	0.4430	1.2605	0.2845

In bold the coefficients significant at 5% level

**Table 11:** F-test for the rest of coefficients of the Model 2A with absolute value of the Spread as independent variable

	Autoregresive		DJI		Gold		EUR		VIX	
Crypto	F-AR	*p*-value	FDJI-AR	*p*-value	FGOLD-AR	*p*-value	FEUR-AR	*p*-value	FVIX-AR	*p*-value
BTC	**14.3671**	**0.0000**	0.5407	0.5827	0.5336	0.4651	0.0674	0.9348	1.1236	0.3391
ETH	**23.6377**	**0.0000**	0.5474	0.5788	1.2657	0.2606	0.3955	0.7563	0.7643	0.3820
USDT	**21.8440**	**0.0000**	1.9251	0.1247	0.7991	0.4504	0.7698	0.4637	**7.2713**	**0.0070**
XRP	**15.6709**	**0.0000**	0.0650	0.7988	1.0035	0.3910	0.7177	0.5418	0.8321	0.4358
LTC	**204.3897**	**0.0000**	2.0556	0.1517	0.3967	0.5288	2.0571	0.1515	0.1886	0.6641
BCH	**8.8588**	**0.0000**	**84,307.0200**	**0.0000**	0.6409	0.4234	**9.0358**	**0.0001**	0.4049	0.5246
ADA	**13.3305**	**0.0000**	0.9597	0.3273	2.5902	0.1075	**5.5008**	**0.0190**	2.0539	0.1056
BNB	**14.8008**	**0.0000**	0.7775	0.4602	0.0006	0.9799	0.8579	0.4629	0.1381	0.7102

In bold the coefficients significant at 5% level

**Table 12:** F-test for the rest of coefficients of the Model 2B with absolute value of the Spread as independent variable

	Autoregresive		DJI		Gold		EUR		VIX	
Crypto	F-AR	*p*-value	FDJI-AR	*p*-value	FGOLD-AR	*p*-value	FEUR-AR	*p*-value	FVIX-AR	*p*-value
BTC	**22.0695**	**0.0000**	0.0927	0.7608	2.3706	0.1236	**4.6745**	**0.0306**	**10.9432**	**0.0009**
ETH	**26.8924**	**0.0000**	0.4820	0.4875	0.7055	0.4010	3.6194	0.0571	**6.6780**	**0.0098**
USDT	**15.2936**	**0.0000**	0.5359	0.4642	0.9698	0.3247	**37.4209**	**0.0000**	**14.3868**	**0.0001**
XRP	**14.0026**	**0.0000**	0.0296	0.8634	0.6332	0.5939	0.5584	0.5725	**6.2249**	**0.0022**
LTC	**204.3897**	**0.0000**	2.0571	0.1515	0.1886	0.6641	**7.5792**	**0.0059**	**7.8361**	**0.0051**
BCH	**165.8747**	**0.0000**	2.4603	0.1168	**6.9832**	**0.0082**	**62.5327**	**0.0000**	**26.7516**	**0.0000**
ADA	**13.4294**	**0.0000**	**4.6706**	**0.0307**	0.1732	0.6773	2.4894	0.0841	**8.5273**	**0.0000**
BNB	**22.1695**	**0.0000**	0.0590	0.8081	1.9702	0.1604	1.2429	0.2895	**15.4177**	**0.0001**

In bold the coefficients significant at 5% level

**Table 13:** F-test for the rest of coefficients of the Model 3A

	Autoregresive		DJI		Gold		EUR		VIX	
Crypto	F-AR	*p*-value	FDJI-AR	*p*-value	FGOLD-AR	*p*-value	FEUR-AR	*p*-value	FVIX-AR	*p*-value
BTC	**67.4294**	**0.0000**	**4.4239**	**0.0044**	**95.1753**	**0.0000**	**48.9249**	**0.0000**	**23.2155**	**0.0000**
ETH	**53.1334**	**0.0000**	**9.1821**	**0.0001**	**4.9829**	**0.0072**	**22.4996**	**0.0000**	**24.7542**	**0.0000**
USDT	**141.0124**	**0.0000**	**7.7410**	**0.0005**	**30.1598**	**0.0000**	0.2481	0.6184	0.7754	0.5082
XRP	**99.5784**	**0.0000**	**17.1688**	**0.0000**	**5.5290**	**0.0042**	1.6976	0.1926	**4.0819**	**0.0071**
LTC	**4.1860**	**0.0408**	2.6016	0.1068	0.3640	0.5463	1.6934	0.1850	0.0007	0.9783
BCH	**28.1819**	**0.0000**	**18.4505**	**0.0000**	**38.6301**	**0.0000**	**46.1253**	**0.0000**	**17.6922**	**0.0000**
ADA	**4.9220**	**0.0007**	1.0770	0.3415	0.3662	0.7774	1.6065	0.2050	0.6982	0.5535
BNB	**22.1580**	**0.0000**	**9.9328**	**0.0016**	2.7014	0.0682	**17.7812**	**0.0000**	**14.2256**	**0.0000**

In bold the coefficients significant at 5% level

**Table 14:** F-test for the rest of coefficients of the Model 3B

	Autoregresive		DJI		Gold		EUR		VIX	
Crypto	F-AR	*p*-value	FDJI-AR	*p*-value	FGOLD-AR	*p*-value	FEUR-AR	*p*-value	FVIX-AR	*p*-value
BTC	1.6653	0.1903	**7.3780**	**0.0001**	**47.5956**	**0.0000**	**40.8525**	**0.0000**	**65.5908**	**0.0000**
ETH	**10.7386**	**0.0000**	**27.2501**	**0.0000**	0.7096	0.5466	**14.5504**	**0.0001**	**8.1750**	**0.0003**
USDT	**115.0870**	**0.0000**	1.1180	0.3278	**27.0431**	**0.0000**	**128.5613**	**0.0000**	**4.8878**	**0.0270**
XRP	**38.7363**	**0.0000**	**32.6480**	**0.0000**	**6.2097**	**0.0004**	**96.8195**	**0.0000**	**54.5894**	**0.0000**
LTC	1.9904	0.1583	0.3700	0.5430	2.0472	0.1525	**26.7123**	**0.0000**	0.3723	0.5417
BCH	**169.6035**	**0.0000**	**15.3798**	**0.0000**	**34.9868**	**0.0000**	**37.5652**	**0.0000**	**11.7475**	**0.0000**
ADA	1.7581	0.1362	0.5367	0.5851	0.1797	0.8356	0.4982	0.6080	0.4737	0.7007
BNB	**702.1545**	**0.0000**	**22.0364**	**0.0000**	**11.3750**	**0.0000**	**7.2391**	**0.0008**	0.3723	0.5417

In bold the coefficients significant at 5% level

**Table 15:** F-test for the rest of coefficients of the Model 3A with absolute value of the Spread as independent variable

	Autoregresive		DJI		Gold		EUR		VIX	
Crypto	F-AR	*p*-value	FDJI-AR	*p*-value	FGOLD-AR	*p*-value	FEUR-AR	*p*-value	FVIX-AR	*p*-value
BTC	**81.5360**	**0.0000**	**5.9144**	**0.0006**	**76.7540**	**0.0000**	**60.7611**	**0.0000**	**23.3762**	**0.0000**
ETH	**77.6548**	**0.0000**	**57.6839**	**0.0000**	**4.8288**	**0.0026**	**21.1146**	**0.0000**	**23.1830**	**0.0000**
USDT	**3766.4130**	**0.0000**	**10.1621**	**0.0000**	**40.9958**	**0.0000**	0.2847	0.5936	2.3916	0.1220
XRP	**90.5673**	**0.0000**	**15.6336**	**0.0001**	**6.0122**	**0.0005**	0.4925	0.4828	**3.1762**	**0.0240**
LTC	2.6966	0.1006	1.5421	0.2150	0.0186	0.9816	**3.0055**	**0.0301**	0.1602	0.8520
BCH	**78.2891**	**0.0000**	2.2588	0.1056	**45.5726**	**0.0000**	**35.7814**	**0.0000**	**8.5544**	**0.0000**
ADA	**5.5222**	**0.0002**	1.4588	0.2336	0.2171	0.8050	1.7103	0.1910	0.3235	0.7238
BNB	**120.3930**	**0.0000**	**8.8204**	**0.0000**	**3.3792**	**0.0183**	**6.5725**	**0.0002**	1.4742	0.2247

In bold the coefficients significant at 5% level

**Table 16:** F-test for the rest of coefficients of the Model 3B with absolute value of the Spread as independent variable

	Autoregresive		DJI		Gold		EUR		VIX	
Crypto	F-AR	*p*-value	FDJI-AR	*p*-value	FGOLD-AR	*p*-value	FEUR-AR	*p*-value	FVIX-AR	*p*-value
BTC	**21.0819**	**0.0000**	2.7581	0.0968	**55.8865**	**0.0000**	**42.3011**	**0.0000**	**33.3390**	**0.0000**
ETH	**56.5534**	**0.0000**	**6.1787**	**0.0004**	0.5761	0.5625	**10.1059**	**0.0001**	**34.9959**	**0.0000**
USDT	**113.5755**	**0.0000**	**8.9064**	**0.0002**	**24.5851**	**0.0000**	**19.0579**	**0.0000**	2.2596	0.1328
XRP	**31.1302**	**0.0000**	**13.5270**	**0.0002**	1.8420	0.1747	0.2864	0.5925	**12.1866**	**0.0005**
LTC	**8.2261**	**0.0000**	**4.4987**	**0.0040**	0.5889	0.6225	0.3588	0.6987	1.7197	0.1803
BCH	**65.5134**	**0.0000**	**46.8933**	**0.0000**	**61.3212**	**0.0000**	**12.4771**	**0.0000**	**17.7495**	**0.0000**
ADA	1.0649	0.3456	0.7978	0.3718	0.1168	0.7325	2.7026	0.1002	0.3137	0.7309
BNB	**101.7393**	**0.0000**	**14.6961**	**0.0000**	1.6053	0.1874	**12.5748**	**0.0000**	**8.3062**	**0.0003**

In bold the coefficients significant at 5% level

## Abbreviations

ARCH: Autoregressive Conditional Heteroskedasticity model
ADA: Cardano
ADF: Augmented Dickey–Fuller
AIC: Akaike Information Criterion
BCH: Bitcoin Cash
BNB: Binance
BTC: Bitcoin
CC: Cryptocurrency
CCs: Cryptocurrencies
DJIA: Dow Jones Industrial Average
ETH: Ethereum
EURUSD: Euro to United States Dollar exchange
GARCH: Generalized Autoregressive Conditional Heteroskedasticity
LTC: Litecoin
USDT: Tether
VIX: CBOE Volatility Index
XRP: Ripple
